# Expression profiles of exosomal tRNA-derived fragments and their biological functions in lipomas

**DOI:** 10.3389/fcell.2022.942133

**Published:** 2022-08-10

**Authors:** Yuxi Zhou, Daixi Tao, Zifei Shao, Xiang Wang, Jinhao Xu, Yiyang Li, Kun Li

**Affiliations:** ^1^ Department of Xiangya Stomatological Hospital and Xiangya School of Stomatology, Central South University, Changsha, Hunan, China; ^2^ Department of Changsha Traditional Chinese Medicine Hospital, Changsha, Hunan, China

**Keywords:** lipoma, exosome, tRNA-derived fragments, expression profile, bioinformatics

## Abstract

There is evidence that exosomes derived from the lipoma tissue (Exo-LT) have a stronger capacity to promote the proliferation and migration of adipose-derived stem cells (ADSCs) than those from the adipose tissue (Exo-AT). But the Exo-LT do not have a significant effect on the adipogenic differentiation of the ADSCs. Recently, certain exosomal tRNA-derived fragments (tRFs) have been shown to play a crucial role in the pathogenesis of certain tumors. Therefore, it is necessary to identify the differently expressed tRFs in Exo-LT to further elucidate their molecular functions in lipomas. High-throughput sequencing was performed to examine the tRFs and mRNAs from the all samples belonging to the Exo-LT and Exo-AT groups. Target prediction and bioinformatics analysis were performed to explore their downstream mRNAs and biological functions. In total, 456 differently expressed tRFs and tiRNAs were identified in the Exo-LT group, 12 of which were up-regulated and 12 were down-regulated, respectively. Notably, tRF-1001 was most obviously down-regulated and tRF-3004a was most obviously up-regulated in the Exo-LT group. Moreover, among the target genes of tRF-1001 and tRF-3004a, both JAG2 and VSIG4 were significantly down-regulated in the Exo-LT group, while WNT5A, COL1A1, and PPARGC1A were highly expressed in both the Exo-LT and Exo-AT groups. The significant down-regulation of JAG2 and VSIG4 in the Exo-LT group could be due to the fact that Exo-LT had a stronger capacity to promote the proliferation and migration of ADSCs compared to the Exo-AT. The high expression of WNT5A, COL1A1, and PPARGC1A in both the Exo-LT and Exo-AT groups could be due to the similar ability of Exo-LT and Exo-AT to promote the adipogenic differentiation of ADSCs.

## Introduction

Lipomas are benign tumors of the adipose tissue, and have an unclear etiology and pathogenesis (Omonte et al., 2016). According to previous reports, numerous adipocytes in the lipoma tissue are surrounded by Ki67+/CD34^+^ cells and exhibit alterations in several biological processes, including proliferation, apoptosis, and stemness (Suga et al., 2009; Zavan et al., 2015). The lipoma tissue has been demonstrated to be an important source of stem cells (Lin et al., 2007; Suga et al., 2009; Tremp et al., 2016). Lipoma-derived stem cells (LDSCs) have been reported to express specific mesenchymal stem cell (MSCs) markers, proliferate in a similar manner as the ADSCs, and could differentiate into adipocytes, chondrocytes, and osteoblasts (Lin et al., 2007; Makiguchi et al., 2013; Tremp et al., 2016). Stojanovic et al. have firstly demonstrated that the mesenchymal phenotypes of LDSCs were similar to that of the ADSCs, but their varying differentiation capacities were governed by distinct molecular signatures (Stojanović et al., 2018). Due to the potential application of lipomas in regenerative medicine, further studies exploring the underlying pathological mechanism of lipomas are urgently needed to ensure that they are not true tumors and could be safely utilized in the clinic.

Exosomes are nano-sized vesicles (30–150 nm in diameter) enclosed by a lipid bilayer (Mathieu et al., 2019), which are released by a variety of cells, such as the endothelial cells, lymphocytes, MSCs, tumor cells, and fibroblasts (Zhang et al., 2017). According to some of the previous multi-omics studies, exosomes carry specific cargo such as proteins, lipids, nucleic acids, and other biomolecules (Subra et al., 2007; Chevillet et al., 2014; Wang et al., 2015). The identified exosomal nucleic acids include coding RNAs and non-coding RNAs, the latter consisting of transfer RNAs (tRNAs), long non-coding RNAs (lncRNAs), and microRNAs (miRNAs), among others (Shurtleff et al., 2017; Wei et al., 2017). The composition of exosomes is quite heterogeneous, depending on its cellular origin and pathological or physiological status, suggesting that the process of cargo recruitment in exosomes is regulated (Conigliaro et al., 2017). Furthermore, exosomes are considered as exhilarating new models for delivering RNA-targeted therapeutics owing to their ability to protect the cargo (including nucleic acids and other biological molecules) from degradation *in vivo* and target them to specific recipient cells (O'Brien et al., 2020).

Adipose tissue is known to actively secrete exosomes, moreover, exosomes derived from the adipose tissue (Exo-AT) have been reported to be the main source of circulating exosomes (Ying et al., 2017). Additionally, it has been confirmed that Exo-AT was involved in numerous biological processes, including the induction of adipogenesis in the ADSCs *in vitro* and promoting adipose tissue regeneration *in vivo* (Dai et al., 2017; Zhang et al., 2017). ADSCs are important functional cells that promote adipogenesis in adipose tissue (Li et al., 2017). Recently, Hong et al. showed that the lipoma tissue could also secrete exosomes, and ADSCs could internalize the exosomes derived from the lipoma tissue (Exo-LT) through endocytosis (Hong et al., 2021). In addition, both Exo-LT and Exo-AT promoted the proliferation, migration, and adipogenic differentiation of ADSCs, with no significant difference in their ability to promote the adipogenic differentiation of ADSCs. However, Exo-LT had a stronger ability to promote proliferation and migration of the ADSCs (Hong et al., 2021). Based on these results, we speculated that the reason for the stronger ability of Exo-LT to promote the proliferation and migration of ADSCs could be due to the specific biomolecules within the Exo-LT, such as non-coding RNAs.

With the advancement of high-throughput sequencing techniques and bioinformatics analysis, recently, a novel small nuclearRNA (snRNA) derived from tRNAs was reported (Yu et al., 2020). These tRNA-derived fragments are broadly classified into tRNA-derived small RNA fragments (tRFs) and tRNA halves (tiRNAs). tRFs are divided into four types according to their location on the transcripts, including tRF-1, tRF-2, tRF-3, and tRF-5 (Kumar et al., 2016; Ma et al., 2020; Qin et al., 2020). tiRNAs are further divided into 3′tiRNAs and 5′tiRNAs (Zhu et al., 2019b; Tao et al., 2020). Moreover, tRFs and tiRNAs have been shown to exert biological effects through multiple mechanisms, such as regulating protein translation, epigenetic modification, controlling cell cycle, and immune signals (Zhu et al., 2019a; Cao et al., 2020; Kim et al., 2020). Notably, studies have shown that tRFs were involved in regulating the proliferation and metastasis of cancer cells, implying that they have great clinical potential in the diagnosis and treatment of different types of cancer (Oberbauer and Schaefer, 2018; Shen et al., 2018; Sun et al., 2018). However, till date, there are no relevant reports on the possible role of tRFs/tiRNAs in lipomas.

Overall, comprehensive and detailed comparisons between the lipoma tissue and adipose tissue is lacking, and to date, no studies have analyzed the differently expressed tRFs/tiRNAs in Exo-LT. Hence, in the current study, we detected the differential expression of tRFs/tiRNAs and mRNAs between the Exo-LT group and the Exo-AT group using high-throughput sequencing. We further explored their potential molecular functions in lipomas through bioinformatics analysis. The above strategy enabled the identification of potentially novel biomarkers for the diagnosis and the prognosis of lipoma patients, new targets for precision therapy of lipomas, as well as new methods to distinguish the lipoma tissue from the adipose tissue.

## Materials and methods

### Clinical specimens

We ensure that all patients were notified of the objective and procedures in our study before surgery and that they agreed to provide the tissue that was removed. At same, we obtained the written consent of each subject participating in this study. Both subcutaneous lipoma tissue and adipose tissue were taken from male patients who received skin transplantation at Xiangya Stomatological Hospital, Changsha, Hunan, China. The three lipoma samples and the three adipose tissue samples were obtained from six different patients. The three normal adipose tissue samples were taken from the patient’s abdomen. Of the three lipoma tissue samples, two were taken from the patient’s abdomen and one was taken from the patient’s back.

### Isolation of Exo-AT and Exo-LT

The collected adipose tissue and lipoma tissue of the patient were soaked in PBS containing 100U/ml penicillin and 100 μg/ml streptomycin for 10 min, and then washed with PBS for 3 times. Then, we cut them into small pieces and added to a Celstir spinner flask (Wheaton) supplied with Dulbecco’s modified eagle medium/f12 (DMEM/F12) individually, and cultured in suspension at 100 rpm and 37°C for 2 days. Cell debris was discarded by centrifugation (2000 g, 3270 rpm, 30 min). Amicon® Ultra-50 Centrifugal Filter with Ultracel-3 membrane (3000Mw, Millipore) was used to centrifuge (5000 g, 4°C, 30 min) and obtain the concentrated adipose tissue extract (ATE) and lipoma tissue extract (LTE). And we add the ATE and LTE to the Total Exosome Isolation™ reagent (Life Technologies, Volume ratio: concentrated solution: kit = 2:1, 4°C, overnight), followed by centrifugation (10,000 g, 4°C, 1 h) to retain the pellet.

### Characterization of Exo-AT and Exo-LT

Transmission electron microscopy (TEM) (FEI Tecnai G2 Spirit, United States) was used to obtain the ultrastructure of the Exo-AT and Exo-LT, and ZetaView® system (Particle Metrix, Germany) was performed to analyze the size distribution of the Exo-AT and Exo-LT. Exo-AT and Exo-LT were added in RIPA Lysis Buffer (KeyGEN, China) and blotted on polyvinylidene fluoride (Millipore). Incubate Exo-AT and Exo-LT samples with primary antibodies, including CD9 (1:1,000, Abcam, ab92726), CD63 (1:1,000, Abclonal, a5271), TSG101 (1:1,000, Abcam, Ab125011), and β-ACTIN (1:1,000, proteintech, 66009-1-ig) at 4°C overnight, and then incubate them with secondary antibody (Rabbit secondary antibody 1:5,000; Mouse secondary antibody 1:10000) at 37°C for 2 h. Visualize and photograph the labeled protein markers by ImageQuant LAS 4000 mini (GE Healthcare).

### Total RNA extraction and library preparation

We used the TRIzol reagent (Thermo Fisher Scientific) to isolate the total RNAs from Exo-AT samples (*n* = 3) and Exo-LT samples (*n* = 3) according to the instructions. Agarose gel electrophoresis (Biotechnology and Biological Engineering Co., Ltd) and Nanodrop™ (Nanodrop, ND-1000) were used to check the integrity and quantity of each RNA sample. We did the following treatments for all RNA samples before the library preparation: 3′-aminoacyl (charged) deacylation to 3′-OH for 3′adaptor ligation, 3′- cyclic phosphate removal to 3′-OH for 3′adaptor ligation, 5′-OH (hydroxyl group) phosphorylation to 5′-P for 5′-adaptor ligation, and m1A and m3C demethylation for reverse transcription. Using an automated gel cutter (Dark Reader, DR-46 B) to select the size of sequencing libraries for the sequencing RNA biotypes. Agilent BioAnalyzer 2,100 (Agilent, China) was used to qualify and quantify the libraries.

### tRF/tiRNA and mRNAs sequencing

The standard small RNA sequencing type on the Illumina NextSeq (Illumina, 500) was 50bp singleread. tRNAs sequences of cytoplasmic were downloaded from GtRNAdb (Chan and Lowe, 2009; Chan and Lowe, 2016) and tRNAs sequences of mitochondrial were forecasted through tRNAscan-SE software (Lowe and Eddy, 1997; Lowe and Chan, 2016). The predicted intronic sequence was removed and an additional 3′-terminal “CCA” was added to each tRNA in order to generate the mature tRNA libraries. Forty nucleotides of flanking genomic sequence of the original tRNAs sequences were included in order to generate the precursor tRNA libraries (Selitsky and Sethupathy, 2015). Besides, we also examined the expression of mRNAs in the same batch of Exo-AT and Exo-LT samples to identify the target genes of candidate tRFs/tiRNAs**.**

### Data analysis

The comprehensive analysis we completed included: QC mapping summary of raw sequencing data, alignment of the sequence mapping and reference genome, identification of tRFs/tiRNAs, different expression analysis of tRFs/tiRNAs, hierarchical clustering heatmap, volcano map, and scatter map to show the significantly differently expressed tRFs/tiRNAs in each group comparison. The tRFs/tiRNAs-seq experiment workflow and tRFs/tiRNAs-seq data analysis workflow was shown in Supplementary Figures S1, S2.

### Bioinformatic analysis

A combination application of TargetScan and Miranda was used to predict the target genes of the candidate tRFs/tiRNAs. Moreover, Metascape was used to analyze the biological functions of mRNAs that co-expressed with the candidate tRFs/tiRNAs. Besides, Gene Ontology (GO) project database and Kyoto Encyclopaedia of Genes and Genomes (KEGG) database were performed to analyze the signal pathways of the candidate tRFs/tiRNAs.

### Statistical analysis

We used the Solexa pipeline v1.8 (Off-Line Base Caller software, v1.8) for image analysis. We assessed the difference in results using a two-tailed Student’s t-test and a two-way analysis of variance (ANOVA), assuming a *p*-value< 0.05 was statistically significant.

## Results

### Characterization of Exo-AT and Exo-LT

**FIGURE 1:**
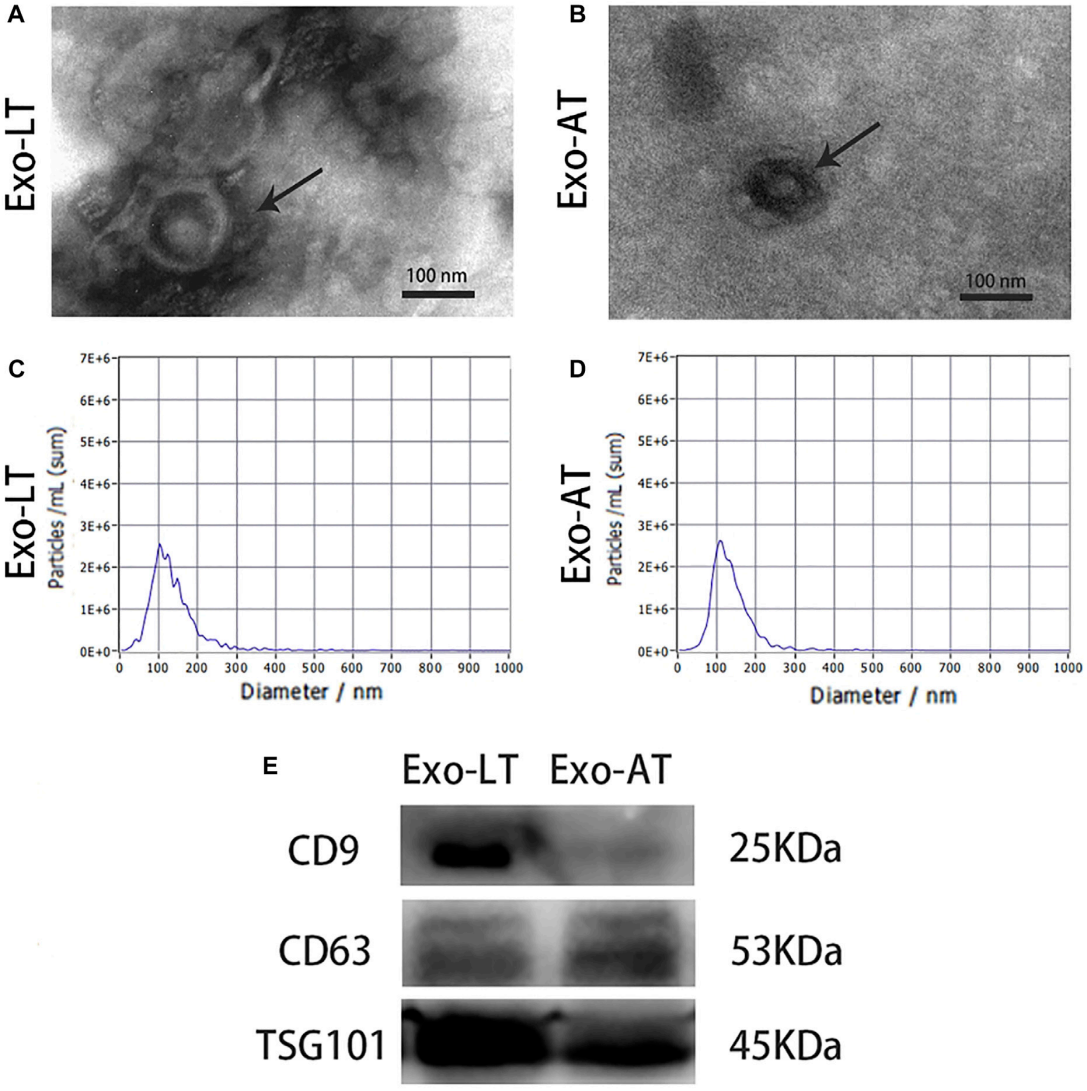
Characterization of Exo-LT and Exo-AT. **(A,B)** The ultrastructure of Exo-LT and Exo-AT under TEM. Scale bar = 100 nm. **(C,D)** The size distribution profile of Exo-LT and Exo-AT by dynamic light scattering. **(E)** Immunoblotting results showing the expression of the Exo-LT and Exo-AT markers, CD9, CD63, and TSG101.

The cup-shaped morphology of Exo-LT and Exo-AT was observed by TEM (Figure 1A). A Particle Metrix Analyzer, called the ZetaView system, was used to track the diameter of the Exo-LT and Exo-AT, while the average diameter of Exo-LT was 121.0 nm and the average diameter of Exo-AT was 123.9 nm (Figure 1B). Western blotting was performed to measure the expression of antigens CD9, CD63, and TSG101, and they were all found to be expressed in both Exo-LT and Exo-AT (Figure 1C). All of the above results were consistent with the defining characteristics of exosomes (Raposo and Stoorvogel, 2013), indicating that Exo-LT and Exo-AT were successfully isolated.

### Expression profiles of tRFs/tiRNAs in Exo-LT and Exo-AT groups

**FIGURE 2:**
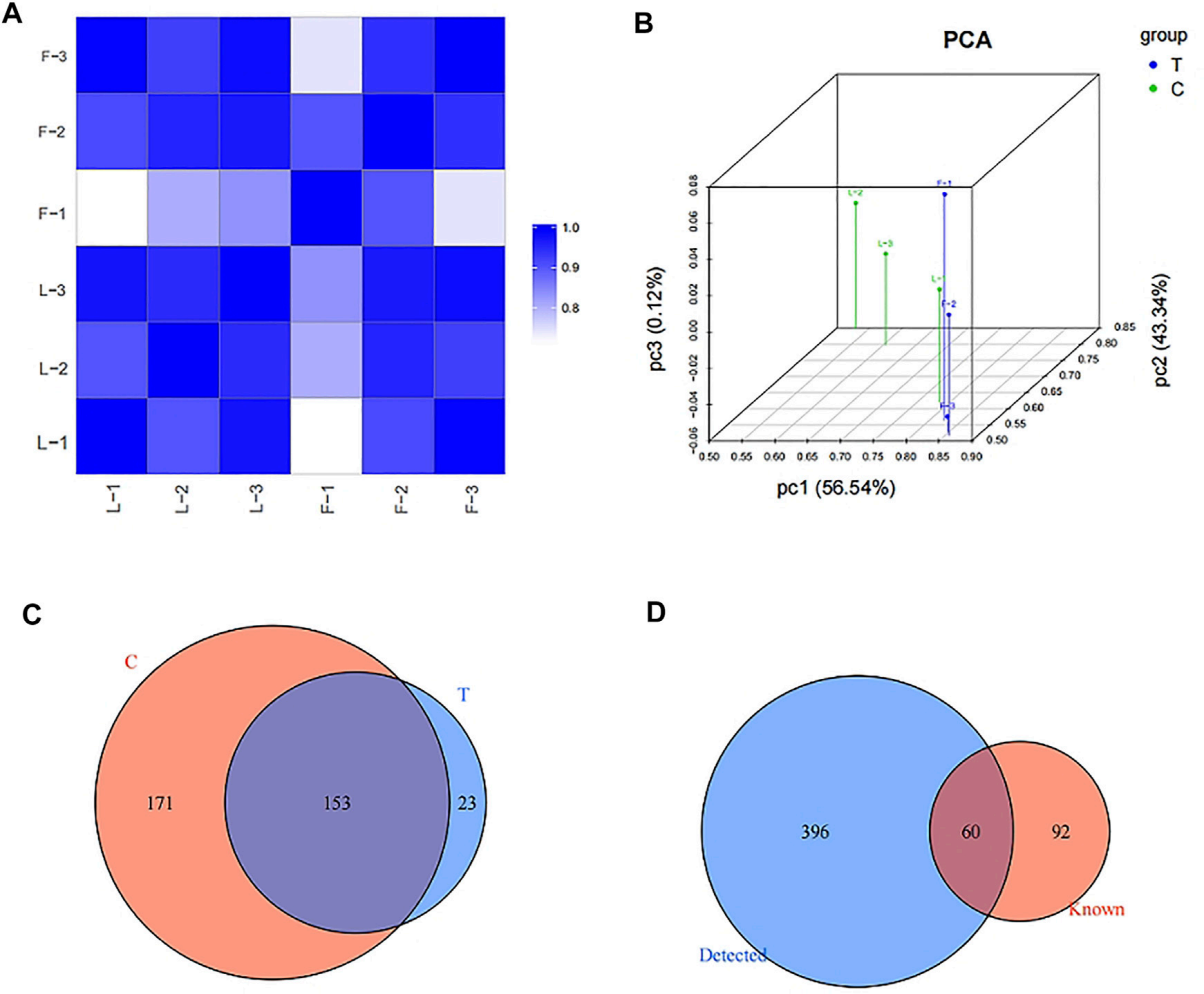
Expression profiles of tRFs/tiRNAs in the Exo-LT and Exo-AT groups. **(A)** Heatmap showing the correlation coefficient of tRFs and tiRNAs from all the samples. Blue represents two samples with high correlation coefficient, while white represents the two samples with low correlation coefficient. **(B)** Principal component analysis (PCA) of tRFs and tiRNAs from all the samples. The colored points represent the corresponding samples, and the space distance represents the similarity in data size. **(C)** Venn diagram based on the number of commonly expressed and specifically expressed tRFs/tiRNAs between the Exo-AT(C) and Exo-LT(T) groups. **(D)** Venn diagram based on the number of known and detected tRFs/tiRNAs differentially expressed between the Exo-AT and Exo-LT groups.

High-throughput RNA-sequencing was conducted to identify the expression levels of tRFs/tiRNAs in both the Exo-LT and Exo-AT groups. Firstly, sequencing quality control for each sample was assessed using the quality (Q) score plots, with a Q score higher than 30 (>99.9% correct) being considered as high-quality data. The tRFs/tiRNAs-seq quality score plots for each sample are shown in Supplementary Figures S3A,F, and the quality score of each sample is listed in Supplementary Table S1. Then, the correlation coefficient was measured depending on the expression level in each sample, and the closer the value was to 1, the more similar the two comparison samples were (Figure 2A). Results from PCA also showed significant differences in the expression profiles of tRFs/tiRNAs across all the samples (Figure 2B). Additionally, the mapping summary of the whole tRFs/tiRNAs is listed in Supplementary Table S2. Finally, a total of 456 dysregulated tRFs/tiRNAs were identified from the two groups, and part of the results for the tRFs/tiRNAs expression profile data are shown in Supplementary Table S3. The Venn diagram revealed that 153 tRFs/tiRNAs were co-expressed in the Exo-LT and the Exo-AT groups, 23 tRFs/tiRNAs were uniquely expressed in the Exo-LT group and 171 tRFs/tiRNAs were uniquely expressed in the Exo-AT group (Figure 2C). Moreover, among these 456 dysregulated tRFs/tiRNAs, 396 were not annotated in the tRFdb database (Kumar et al., 2015) and were identified as new tRFs/tiRNAs (Figure 2D).

**FIGURE 3:**
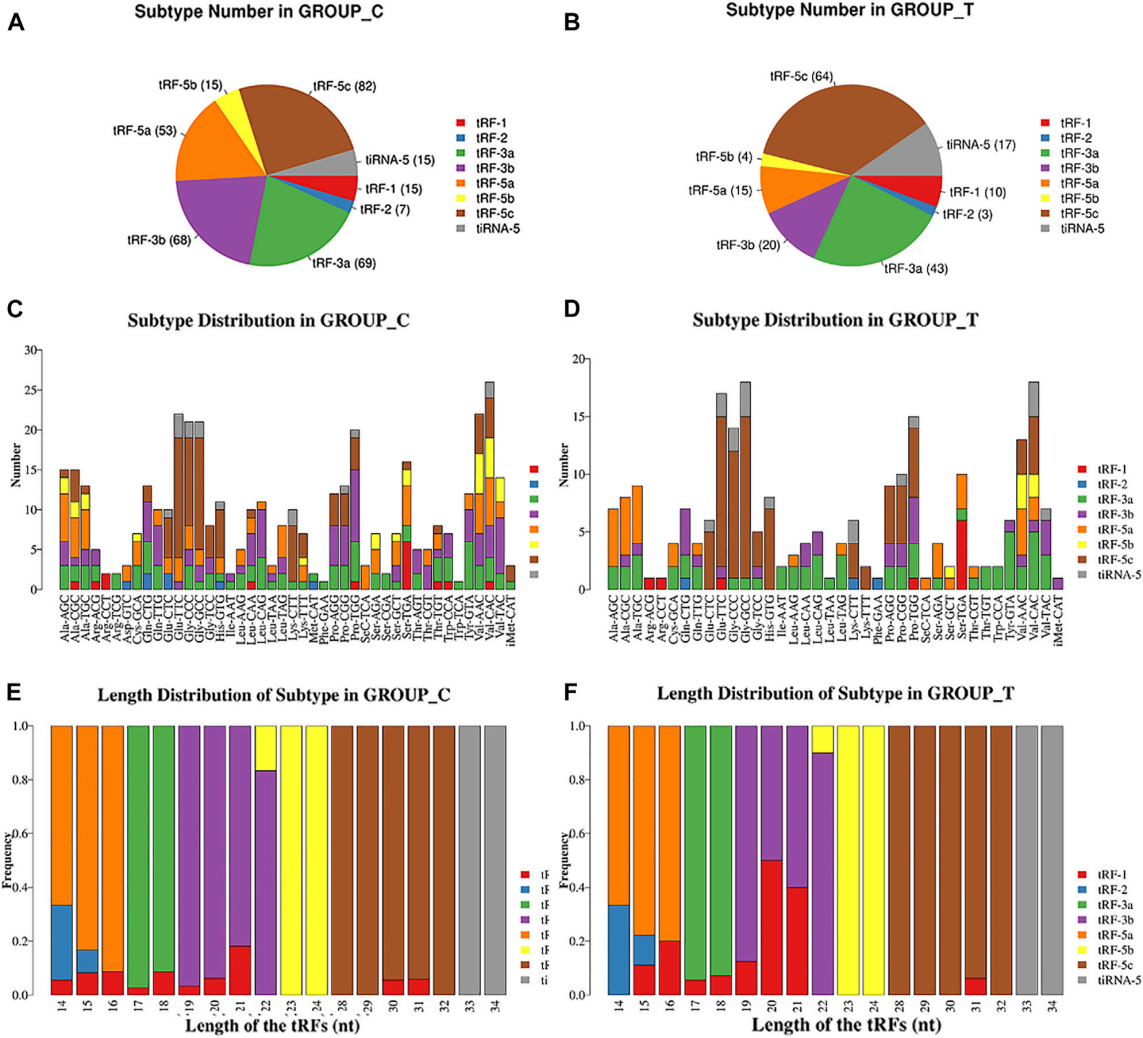
Pie chart and stacked bar chart. **(A,B)** Pie chart of tRFs/tiRNAs isoform distribution between the Exo-AT(C) and Exo-LT(T) groups. The values in brackets represent the number of tRFs/tiRNAs subtypes. **(C,D)** Stacked bar chart for all subtypes of tRFs/tiRNAs expressed in the Exo-AT(C) and the Exo-LT(T) groups clustered by the anticodon of the tRNAs. The X-axis represents the number of all the subtypes of tRNAs derived from the same tRNA anticodon, and the Y-axis shows the tRNAs with the same anticodon. **(E,F)** Stacked bar chart of the frequency of subtypes against the length of the tRFs/tiRNAs in the Exo-AT(C) and the Exo-LT(T) groups. The X axis represents the length of tRFs/tiRNAs and the Y axis shows the frequency of the subtype against the length of the tRFs/tiRNAs. Besides, the bar with color represents the number of each subtype of tRF/tiRNA. The red, blue, green, purple, orange, yellow and brown colors represent the subtypes of tRFs and the gray color represents tiRNA-5.

Since tRFs and tiRNAs are generally classified into subtypes based on their length and cleavage site, we examined the differences in the distribution of tRFs/tiRNAs subtypes in both the Exo-LT and Exo-AT groups, and found highly different expression levels of each of the tRFs/tiRNAs subtypes. In summary, compared with the Exo-AT group, the expression level of tiRNA-5 was higher and the expression levels of the remaining subtypes were decreased in the Exo-LT group (Figures 3A,B). Since the tRFs/tiRNAs sharing the same anticodon could originate from different tRNAs precursors, stacked bar plots were used to compare the number of tRFs/tiRNAs isoforms against the number of tRNAs isodecoders (Figures 3C,D), and the frequency of tRFs/tiRNAs isoforms were compared against their length (Figures 3E,F). The bar charts show the total read counts against the lengths of the trimmed reads (Supplementary Figures S4A,F).

### tRF-1001 and tRF-3004a were differentially expressed in the Exo-LT group

**FIGURE 4:**
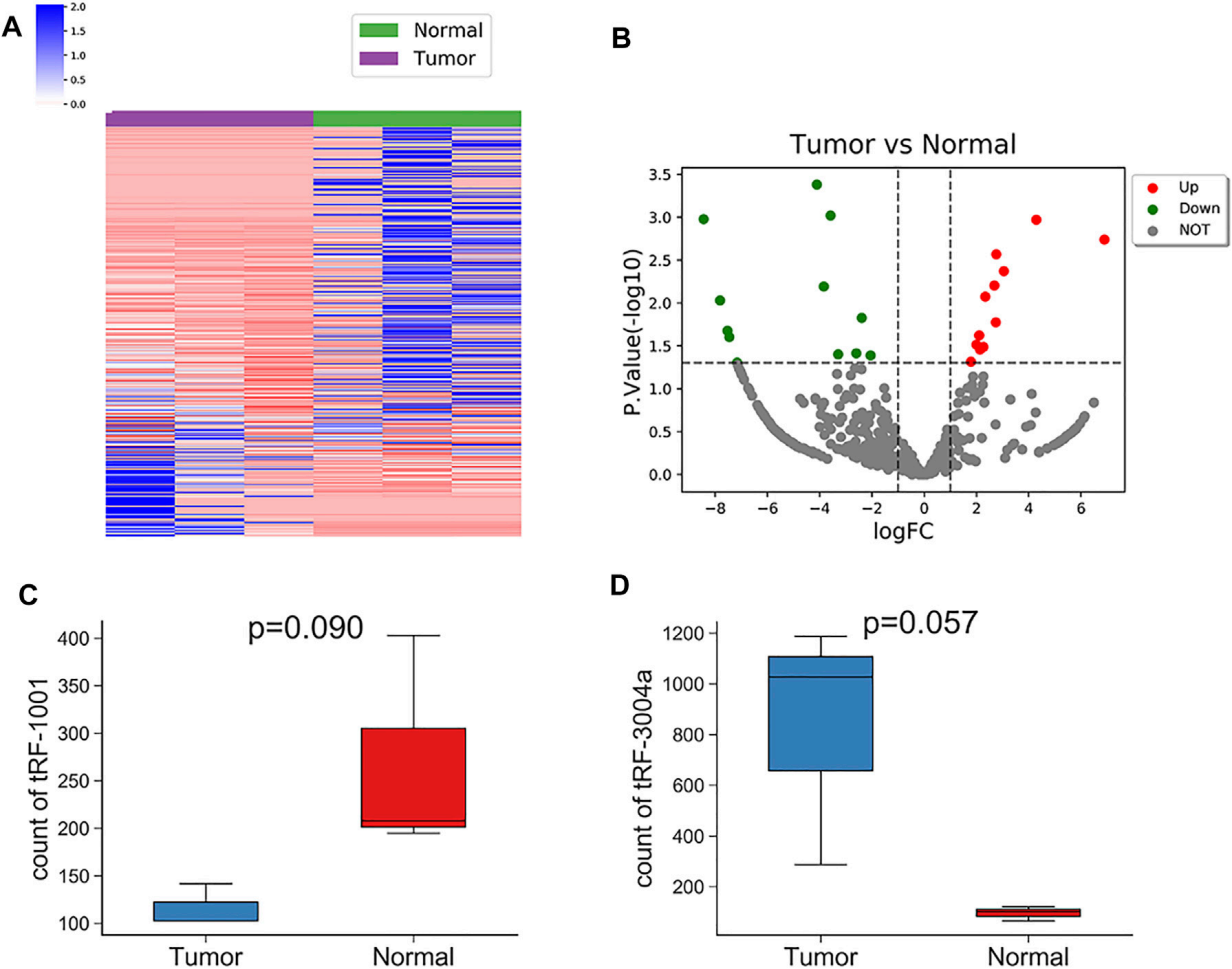
Hierarchical clustering and the differently expressed tRFs/tiRNAs between the Exo-LT(T) and the Exo-AT(N) groups. **(A)** Unsupervised hierarchical clustering heatmap of tRFs/tiRNAs for the Exo-AT and the Exo-LT groups. The blue color represents an expression level below the mean, and red color represents an expression level above the mean. The colored bar on the left side of the panel indicates the division that was performed by K-means. **(B)** Volcano plot of the tRFs/tiRNAs from the Exo-AT and the Exo-LT groups. The red/green circles indicate statistically significant differentially expressed tRFs/tiRNAs with fold change not less than 1.5 and *p*-value ≤ 0.05 (red: up-regulated; green: down-regulated). The gray circles indicate non-differently expressed tRFs/tiRNAs with FC and/or q-value that did not meet the cut-off thresholds. **(C)** Boxplot of the tRF-1001 expression levels in the Exo-AT and the Exo-LT groups. **(D)** Boxplot of the tRF-3004a expression levels in the Exo-AT and the Exo-LT groups. The top and bottom line segments represent the maximum and the minimum values of the data, respectively, and the thick line segment in the middle represents the median of the data.

Based on the fold change difference analysis, 24 tRFs/tiRNAs with significantly different expression (|logFC|>1, *p* < 0.05) were screened out from the 456 dysregulated tRFs/tiRNAs, including 12 up-regulated tRFs/tiRNAs and 12 down-regulated tRFs/tiRNAs. The list of these 24 tRFs/tiRNAs along with the difference in their expression levels between Exo-LT and Exo-AT groups is shown in Supplementary Table S4. The hierarchical clustering plot showed differential tRFs/tiRNAs expression profiles across all the samples (Figure 4A). The volcano plot and scatter plot revealed large differences between the Exo-LT and Exo-AT groups, which was also statistically significant (). Additionally, the sequencing results showed that among the annotated tRFs in the tRFdb database, there were two tRFs, namely, tRF-1001 and tRF-3004a, which were of significance. tRF-1001 was considerably down-regulated in the Exo-LT group (Figure 4C), while tRF-3004a was markedly up-regulated in the Exo-LT (Figure 4D) group. Therefore, we focused our research on these two tRFs.

### Identification of tRF-1001 and tRF-3004a in the Exo-LT group

**FIGURE 5:**
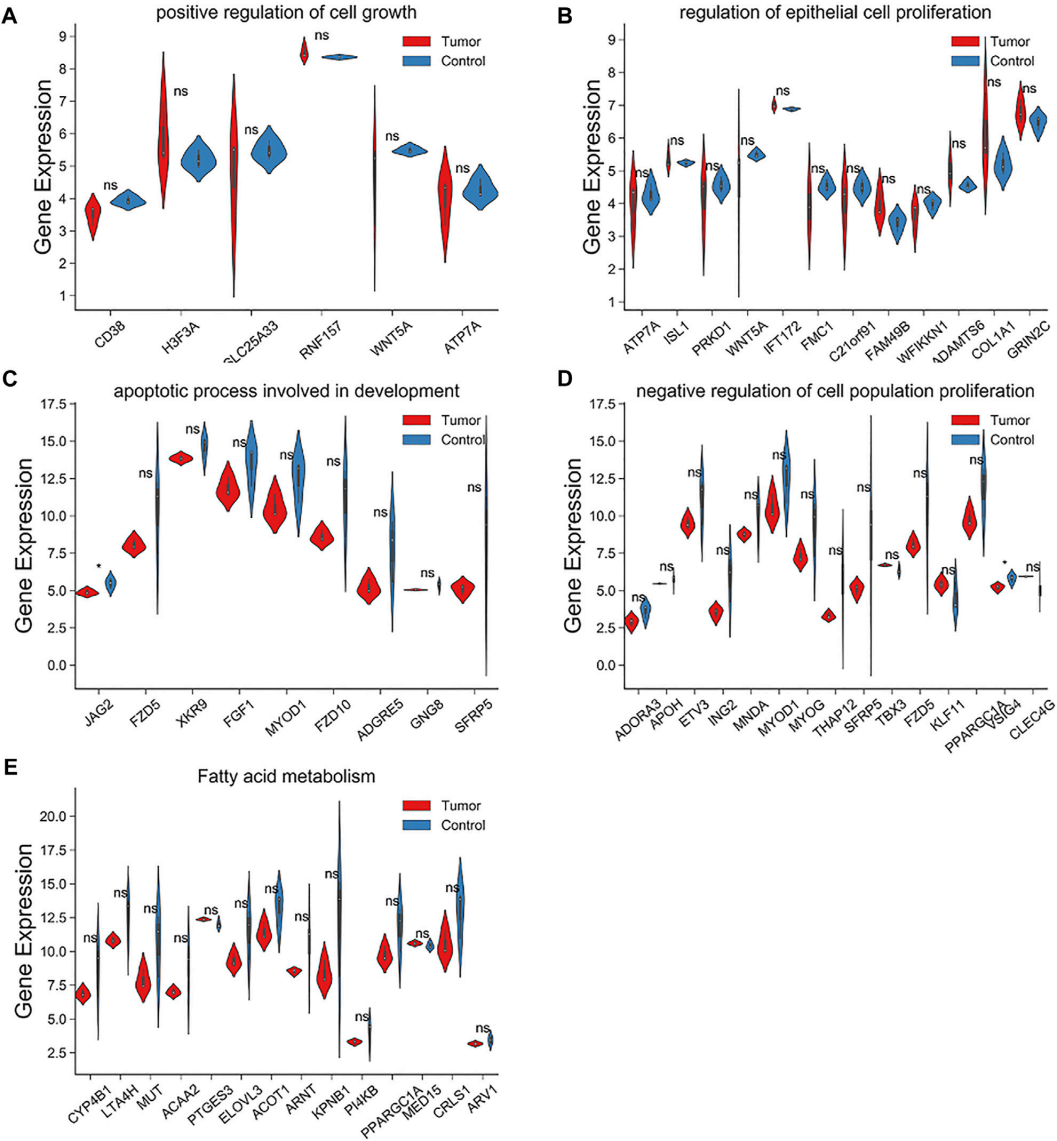
Expression of the tRF-1001 and tRF-3004a target genes in the Exo-LT(T) and the Exo-AT(C) groups. **(A,B)** Gene expression of the target genes of tRF-1001 which positively regulated cell growth pathway and the epithelial cell proliferation pathway. **(C–E)** Gene expression of the target genes of tRF-3004a that were involved in the apoptotic process and in developmental pathway, and in the negative regulation of cell proliferation and fatty acid metabolism.

Within the target genes of tRF-1001 and tRF-3004a, its pathways and signals were closely related to core biological activities, including fatty acid metabolism, cell proliferation, apoptosis and cell growth, which are shown in Table 1 (Table 1). Further analysis revealed that factors involved in the pathways related to tRF-1001 target genes were not significantly dysregulated in the Exo-LT group. Nevertheless, WNT5A, involved in the positive regulation of cell growth pathway, and COL1A1, involved in the regulation of epithelial cell proliferation pathway, were highly expressed in both the Exo-LT and Exo-AT groups (Figures 5A,B). Among the tRF-3004a target genes-related pathways, both JAG2, involved in the apoptotic process and development pathway, and VSIG4, involved in the negative regulation of cell proliferation, were significantly down-regulated in the Exo-LT group (Figures 5C,D). Besides, PPARGC1A, involved in fatty acid metabolism, was highly expressed in both the Exo-LT and Exo-AT groups (Figure 5E).

**TABLE 1:** Core functions and pathway factors of tRF-3004a and tRF-1001 target genes.

tRFs	Term	Description	*p*-value	Symbols
tRF-1001	GO:0030307	positive regulation of cell growth	0.000814	CD38, H3-3A, SLC25A33, RNF157, WNT5A, ATP7A
	GO:0050678	regulation of epithelial cell proliferation	0.00042	ATP7A, ISL1, PRKD1, WNT5A, IFT172, FMC1, C21orf91, CYRIB, WFIKKN1, ADAMTS6, COL1A1, GRIN2C
	GO:1902742	apoptotic process involved in development	0.00162	JAG2, FZD5, XKR9, FGF1, MYOD1, FZD10, ADGRE5, GNG8, SFRP5
tRF-3004a	GO:0008285	negative regulation of cell population proliferation	0.00132	ADORA3, APOH, ETV3, ING2, MNDA, MYOD1, MYOG, THAP12, SFRP5, TBX3, FZD5, KLF11, PPARGC1A, VSIG4, CLEC4G
	R-HSA-8978868	Fatty acid metabolism	0.000493	CYP4B1, LTA4H, MMUT, ACAA2, PTGES3, ELOVL3, ACOT1, ARNT, KPNB1, PI4KB, PPARGC1A, MED15, CRLS1, ARV1

### Bioinformatics analysis of tRF-1001 and tRF-3004a in lipomas

**FIGURE 6:**
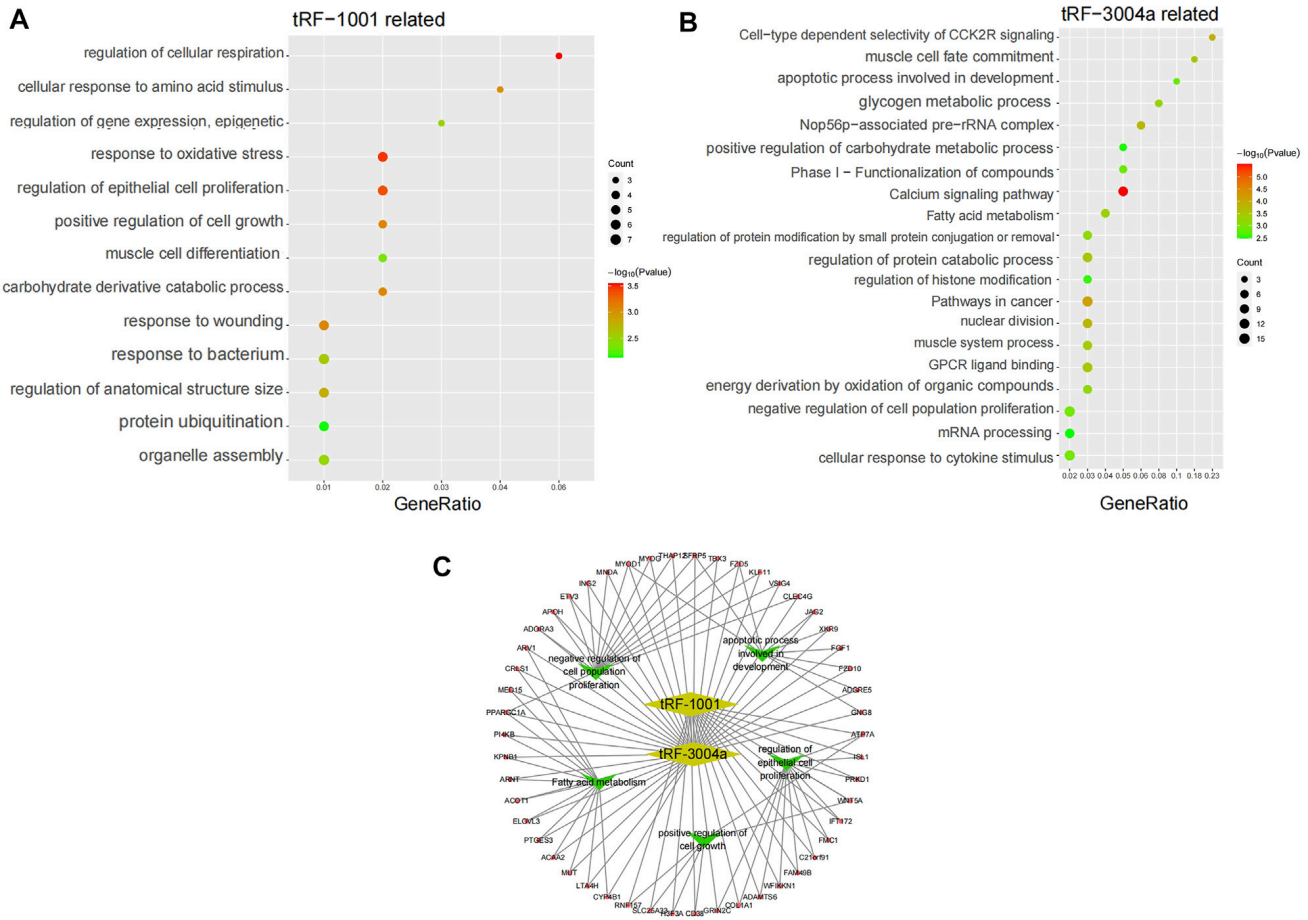
KEGG pathway analysis and pathways network. **(A)** KEGG pathway analysis of tRF-1001 target genes. The vertical axis shows the annotated functions of the target genes. The horizontal axis represents the enrichment score (−log10 transformed *p*-value) and the gene number of each cluster, respectively. **(B)** KEGG pathway analysis of tRF-3004a target genes. **(C)** Network map of the core biological pathways and target genes of tRF-1001 and tRF-3004a.

Amongst the same batch of Exo-LT and Exo-AT samples, we also detected the expression of 18,129 mRNAs in total. Through Pearson correlation analysis, the 78 mRNAs that were significantly co-expressed with tRF-1001, and 236 mRNAs that were significantly co-expressed with tRF-3004a, were screened out according to the threshold (|r|>0.6, *p* < 0.01). Afterwards, the biological functions associated with these mRNAs were separately analyzed using Metascape online. It was found that the 78 mRNAs that were significantly co-expressed with tRF-1001, were mainly involved in cellular respiration, oxidative stress, epithelial cell proliferation, injury regulation, cell growth regulation, cellular response to amino acid stimulation, gene expression regulation, muscle cell differentiation, organelle assembly, protein ubiquitination and other biological functions (Figure 6A). And the 236 mRNAs that were significantly co-expressed with tRF-3004a, were mainly involved in calcium signaling pathway, nuclear division, protein catabolism, fatty acid metabolism, glycogen metabolism, cytokine stimulation response, negative regulation of cell proliferation, apoptosis, mRNA processing and other biological functions (Figure 6B). Furthermore, we constructed an integrated network map of these target genes and core biological function pathways of tRF-1001 and tRF-3004a (Figure 6C).

**FIGURE 7:**
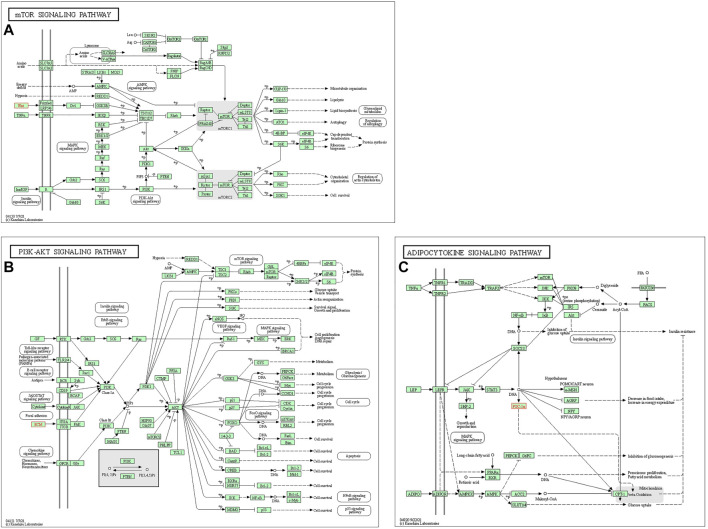
Mapping of the signaling pathways. **(A)** mTOR signaling pathway related to WNT5A. **(B)** PI3K-AKT signaling pathway related to COL1A1. **(C)** ADPOCYTOKINE signaling pathway related to PPARGC1A.

In the end, the signaling pathways associated with WNT5A, COL1A1, and PPARGC1A were listed as the mTOR signaling pathway (Figure 7A), PI3K-AKT signaling pathway (Figure 7B) and ADPOCYTOKINE signaling pathway (Figure 7C), respectively. Moreover, the AMPK signaling pathway associated with PPARGC1A (Supplementary Figures S6A) and the WNT signaling pathway associated with WNT5A (Supplementary Figures S6B) were also displayed.

## Discussion

Currently, with the application of high-throughput sequencing and the advances in bioinformatics techniques, increasing number of dysregulated exosomes tRFs have been reported to be associated with different diseases. It has been reported that tRF-18, tRF-25, and tRF-38 were elevated in plasma exosomes in osteoporosis patients, which showed better diagnostic accuracy for detecting osteoporosis (Zhang et al., 2018). Yang et al. firstly showed that exosomal tRF-1 was significantly up-regulated in serum from systemic lupus erythematosus (SLE) patients, which could serve as a non-invasive biomarker for the prediction and diagnosis of nephritis in SLE patients (Yang et al., 2021). Furthermore, Meng et al. reported the tRFs profile from atopic dermatitis (AD) patients, and suggested that plasma derived exosomal tRF-28 could serve as a potential biomarker for pediatric patients with AD (Meng et al., 2021). In conclusion, different types of exosomal tRFs have been reported as potential diagnostic and predictive biomarkers for various diseases, however, so far, no study has investigated the potential biological functions of tRFs/tiRNAs in Exo-LT or Exo-AT.

Substantial studies have shown the specific functions of miRNAs in the Exo-AT. Adipose tissue has been reported to be the main source of miRNAs in circulating exosomes. Moreover, these adipose-derived circulating miRNAs could act as the regulators of mRNA translation and metabolism in other tissues (Thomou et al., 2017). Adipose-derived macrophages have been shown to release exosomes containing miR-155 that migrated to insulin target cells and regulated insulin sensitivity, insulin response, and glucose homeostasis (Ying et al., 2017). Moreover, Zhang et al. analyzed the miRNAs from both the exosomes derived from the adipose tissues and ADSCs and found that 45 miRNAs were highly expressed in Exo-AT. Interestingly, 14 of them were involved in adipogenesis (Zhang et al., 2017). Until now, there are no studies on tRFs/tiRNAs in the Exo-AT or Exo-LT, and we speculate that tRFs/tiRNAs in Exo-LT might play crucial roles in lipomas and their molecular functions are worth further exploration.

In this study, the RNA sequencing results showed that tRF-1001 was obviously down-regulated and tRF-3004a was clearly up-regulated in the Exo-LT group. tRF-1001 has been reported to be a novel small RNA produced by the cleavage of tRNA precursors in the cytoplasm, which is essential for cell viability and proliferation (Lee et al., 2009). Additionally, tRF-1001 has been reported to be highly expressed in various cancer cell lines, and its expression in the cell lines has been shown to be closely related to cell proliferation (Lee et al., 2009). However, there are no relevant reports on tRF-3004a so far. Therefore, we proposed that the differential expression of tRF-1001 and tRF-3004a in Exo-LT could be related to the differential ability of lipoma and adipose tissue to promote the proliferation and adipogenic differentiation of ADSCs. Therefore, we decided to further investigate its biological functions as well as associated signaling pathways. Currently, studies on tRFs/tiRNAs are not as advanced as those on non-coding RNAs, which has caused a major delay in the construction of tRFs/tiRNAs database. Furthermore, very limited reports on tRF-1001 and tRF-3004a also greatly limit our understanding of their biological functions.

Nevertheless, it has been reported that some tRFs/tiRNAs could bind to the 3′-UTR of specific mRNAs, thereby inhibiting the expression of endogenous targets and suppressing their translation, like miRNAs (Maute et al., 2013; Deng et al., 2015). Thus, to further validate the expression of tRF-1001 and tRF-3004a in lipomas and analyze their biological functions, we also examined the expression of mRNAs in the same batch of Exo-LT samples. A total of 18,129 mRNAs were detected, of which 78 mRNAs were found to be significantly co-expressed with tRF-1001, and 236 mRNAs were significantly co-expressed with tRF-3004a. Then, the biological functions associated with these mRNAs were analyzed using Metascape and we found that the 78 mRNAs significantly co-expressed with tRF-1001 were mainly involved in epithelial cell proliferation, cell growth regulation and other biological functions. Moreover, the 236 mRNAs significantly co-expressed with tRF-3004a were mainly involved in fatty acid metabolism, negative regulation of cell proliferation and other biological functions. It could be seen that tRF-1001 and tRF-3004a in Exo-AT and Exo-LT were closely associated with important intracellular processes such as cell proliferation, growth and fatty acid metabolism. Moreover, the mRNA expression of the Exo-LT group and the Exo-AT group was also significantly different, and both groups highly expressed some mRNAs related to cell proliferation and adipogenesis, the potential impact of differentially expressed mRNAs on lipoma biological functions is currently still under analysis.

Amongst all the target genes of tRF-1001 and tRF-3004a, the target genes JAG2 and VSIG4, of tRF-3004a, were significantly down-regulated in Exo-LT. Previously, JAG2/Notch2 axis has been reported to alleviate intervertebral disc degeneration (IVDD) by modulating apoptosis, proliferation, and the extracellular matrix of nucleus pulposus cells (Long et al., 2019). A significant study has demonstrated that MSCs could escape apoptosis by driving mature dendritic cells to differentiate into JAG2-dependent regulatory dendritic cells (Zhang et al., 2009). Additionally, Zhu et al. revealed that the immuneregulatory activity of ADSCs was somewhat dependent on JAG2 and could become stronger after its multi-differentiation or due to the influence of inflammatory factors (Xishan et al., 2015). VSIG4 has been identified as a novel biomarker of aged murine adipose tissue (Hall et al., 2020). Moreover, VSIG4 has been reported to be markedly down-regulated in fatty livers in patients with nonalcoholic fatty liver disease and in obese mice, whereas the loss of VSIG4 has been shown to accelerate lipid deposition, fibrosis and the severity of inflammatory responses (Li et al., 2019). Overall, the significant down-regulation of JAG2 and VSIG4 in Exo-LT represents decreased apoptosis and increased proliferation, which may be because Exo-LT has stronger effects on the proliferation and migration of ADSCs as compared with Exo-AT.

Interestingly, we also noted in our analysis that the target genes WNT5A and COL1A1 of tRF-1001, and the target gene PPARGC1A of tRF-3004a, were highly expressed in both the Exo-LT and Exo-AT groups. WNT5A has been reported to be involved in the regulation of adipogenesis by multiple studies. Additionally, the microvesicle-derived miR-148a secreted by adipocytes has been shown to regulate osteogenic and adipogenic differentiation by targeting the Wnt5A/Ror2 axis (Duan et al., 2021). It has been reported that COL1A1 played a role in the proliferation, metastasis and invasion of oral squamous cell carcinoma cells (He et al., 2018). PPARGC1A, the master regulator of mitochondrial genesis has been shown to be indispensable during the brown adipocyte formation (Wang et al., 2019). Therefore, the high expression of WNT5A, COL1A1, and PPARGC1A in both the Exo-LT and Exo-AT groups could be related to the insignificant differences between Exo-LT and Exo-AT in promoting the adipogenic differentiation of ADSCs, which is known to enhance the cellular activity and proliferation capacity of ADSCs, requiring the synthesis of large amounts of collagen.

The lipoma samples and adipose tissue samples selected for this study were obtained from different patients because the adipose tissue of lipoma patients may carry genes from the lipoma tissue and confound the sequencing results. The adipose tissue samples we selected were all taken from subcutaneous fat, which belongs to the uniform attribute of yellow fat. However, studies have shown that adipose tissue from different locations of the body, even though they all are subcutaneous, have been shown to have differential gene expression (Rehrer et al., 2012). Therefore, subcutaneous adipose tissue samples taken from different sites may have some influence on the experiment. Furthermore, the above results highlight the significance of tRF-1001 and tRF-3004a in lipomas, however, their specific regulatory roles in the pathogenesis of lipomas need to be further explored in depth. Therefore, experiments investigating protein function are vital to identify the downstream mechanism, and further animal experiments are needed to validate the functions of tRF-1001 and tRF-3004a as therapeutic targets in lipomas. Moreover, it is important to amplify the sample and simultaneously test the levels of tRF-1001 and tRF-3004a in the serum to confirm their feasibility as diagnostic markers. In the sequencing results of this study, the differences in exosomal mRNA expression in the Exo-LT group and Exo-AT group are still under analysis. Last but not least, the current bioinformatics databases do not provide information about tRFs/tiRNAs-microRNAmRNA networks to accurately predict the targets of tRFs/tiRNAs, thus, it is crucial to establish a more comprehensive database to enable better identification and analysis of the biological functions of tRFs/tiRNAs.

This study identified 456 dysregulated tRFs/tiRNAs in the Exo-LT group as compared to the Exo-AT group, of which 12 were significantly down-regulated and 12 were significantly up-regulated. Among them, we found that the expression of tRF-3004a was obviously up-regulated and the expression of tRF-1001 was clearly down-regulated in the Exo-LT group. Additionally, the possible target genes and signaling pathways of tRF-1001 and tRF-3004a were revealed, among which, both JAG2 involved in the apoptotic process and the developmental pathways and VSIG4 involved in the negative regulation of cell proliferation were significantly down-regulated in the Exo-LT group, which could be related to the stronger effects of Exo-LT on the proliferation and migration of ADSCs as compared to Exo-AT. Therefore, both tRF-1001 and tRF-3004a from the Exo-LT group might play an important role in lipomas through multiple biological pathways, which could serve as clinical biomarkers and therapeutic targets for lipomas, as well as a novel method to distinguish lipomas from the adipose tissue. However, the specific molecular mechanisms related to the functional role of tRFs/tiRNAs in Exo-LT and lipomas deserve further in-depth investigation.

## Data Availability

The datasets presented in this study can be found in online repositories. The names of the repository/repositories and accession number(s) can be found in the article/Supplementary Material.

## Abbreviations

Exo-LT, exosomes from lipoma tissue; ADSCs, adipose-derived stem cells; MSCs, mesenchymal stem cells; Exo-AT, exosomes from adipose tissue; tRFs, tRNA-derived fragments; LDSCs, Lipoma-derived stem cells; miRNAs, microRNAs; lncRNAs, long non-coding RNAs; tRNAs, transfer RNAs; tiRNAs, tRNA halves; DMEM/F12, Dulbecco’s modified eagle medium/f12; ATE, adipose tissue extract; LTE, lipoma tissue extract; TEM, transmission electron microscopy; GO, Gene Ontology; KEGG, Kyoto Encyclopaedia of Genes and Genomes; ANOVA, analysis of variance; SLE, systemic lupus erythematosus; IVDD, Intervertebral disc degenerationto; AD, atopic dermatitis.
