# Supplementary figures and images for: Expression profiles of exosomal tRNA-derived fragments and their biological functions in lipomas
Source: Front Cell Dev Biol. 2022 Aug 10;10:942133. doi: 10.3389/fcell.2022.942133 (PMC9399354; doi:10.3389/fcell.2022.942133)

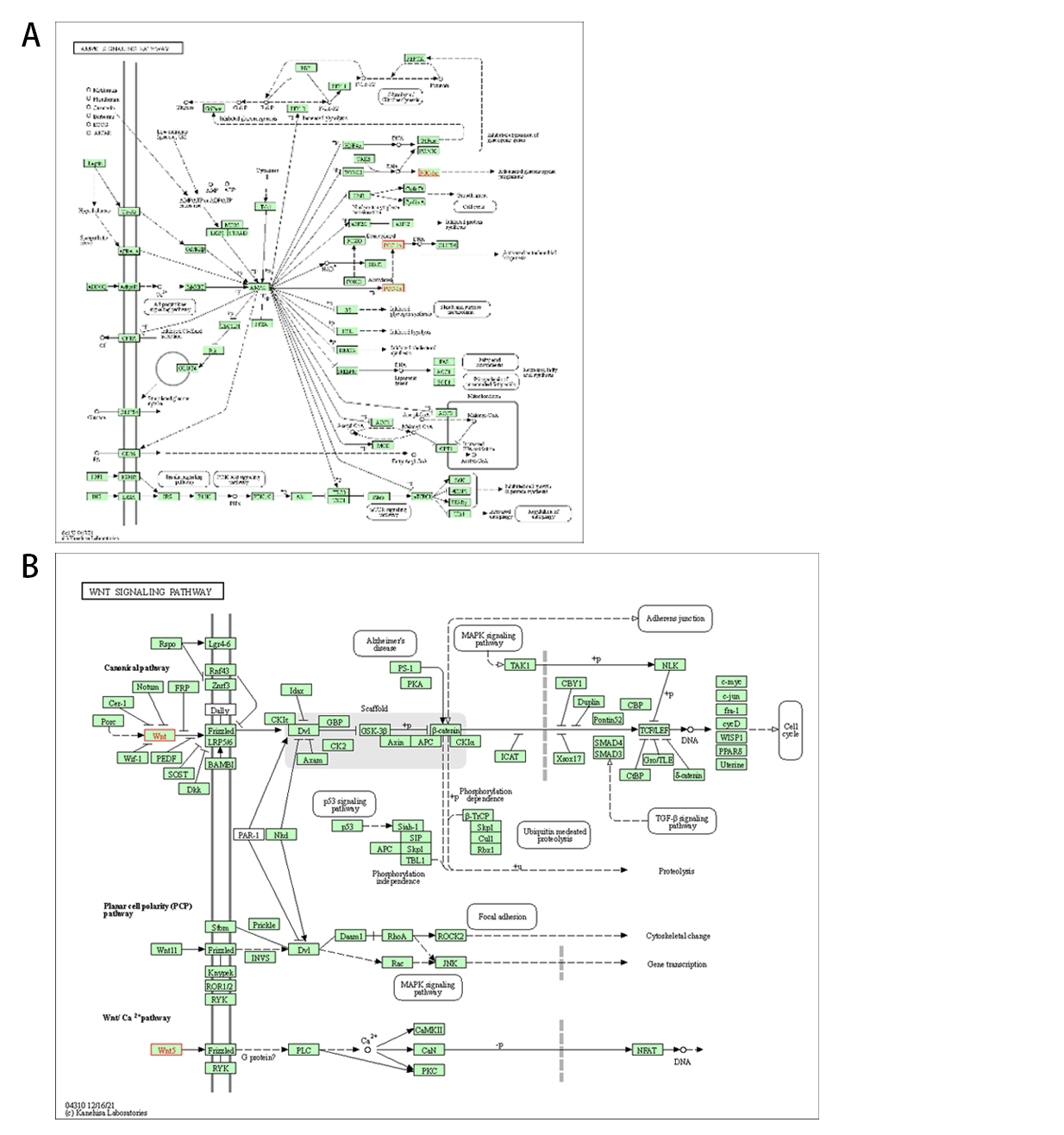

Supplement: Supplementary file 1 [file Image6.jpg]

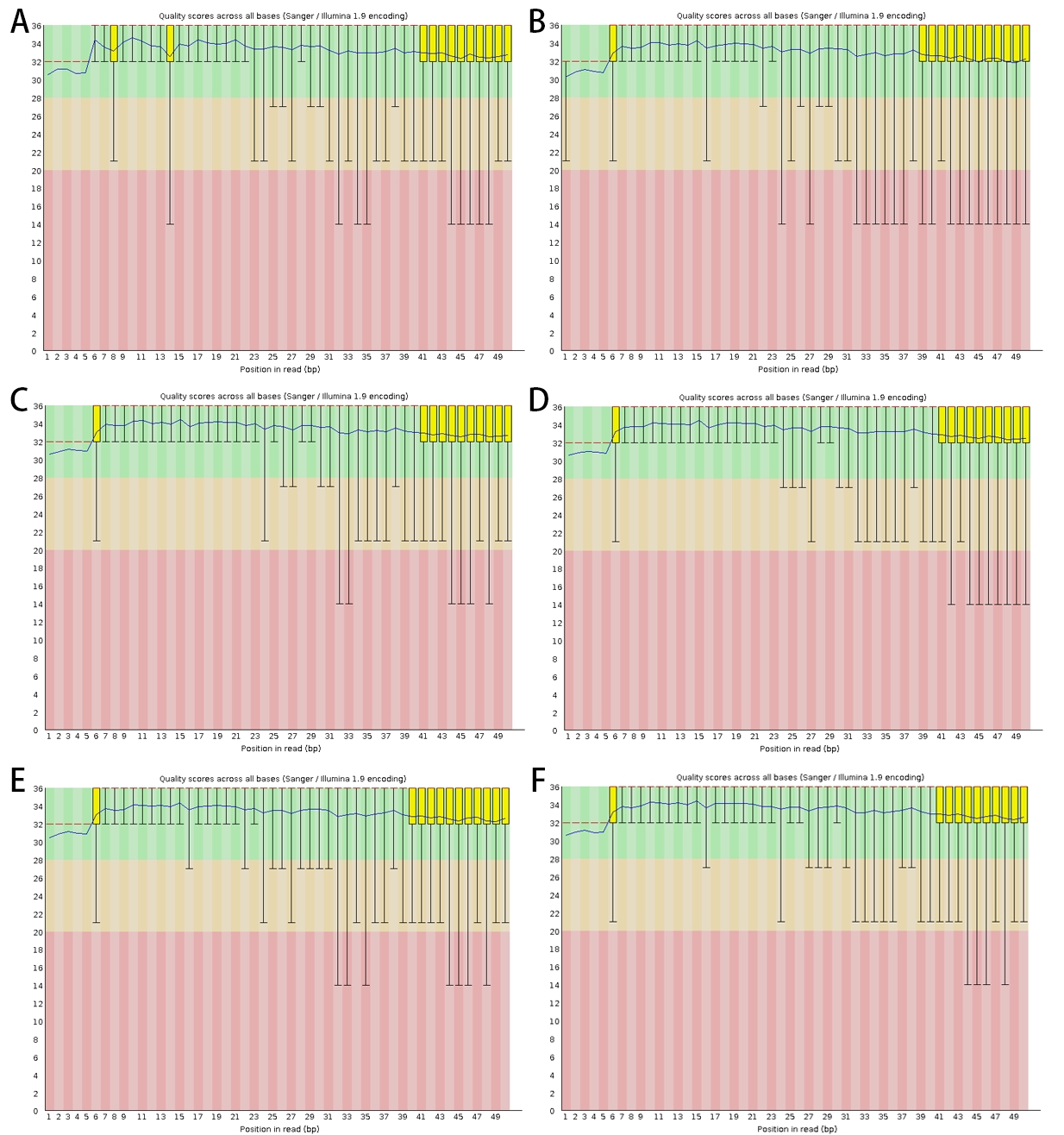

Supplement: Supplementary file 2 [file Image3.jpg]

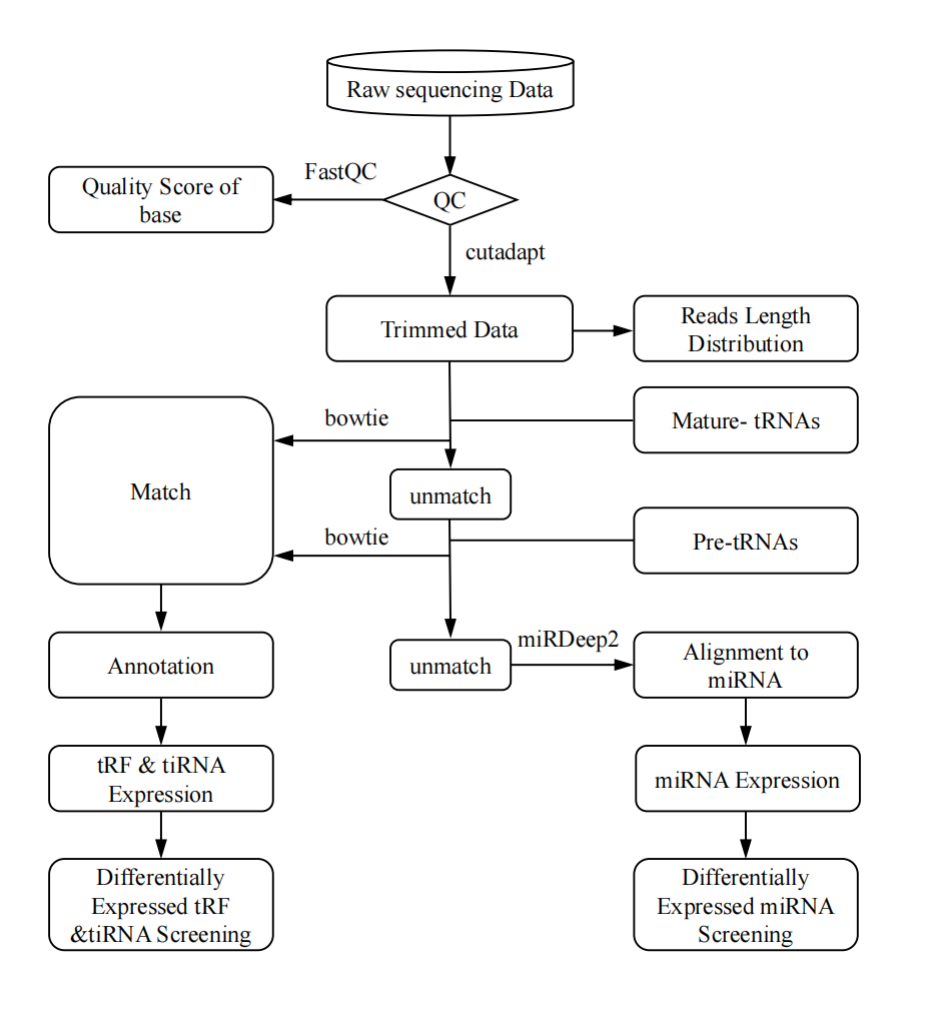

Supplement: Supplementary file 4 [file Image2.tif]

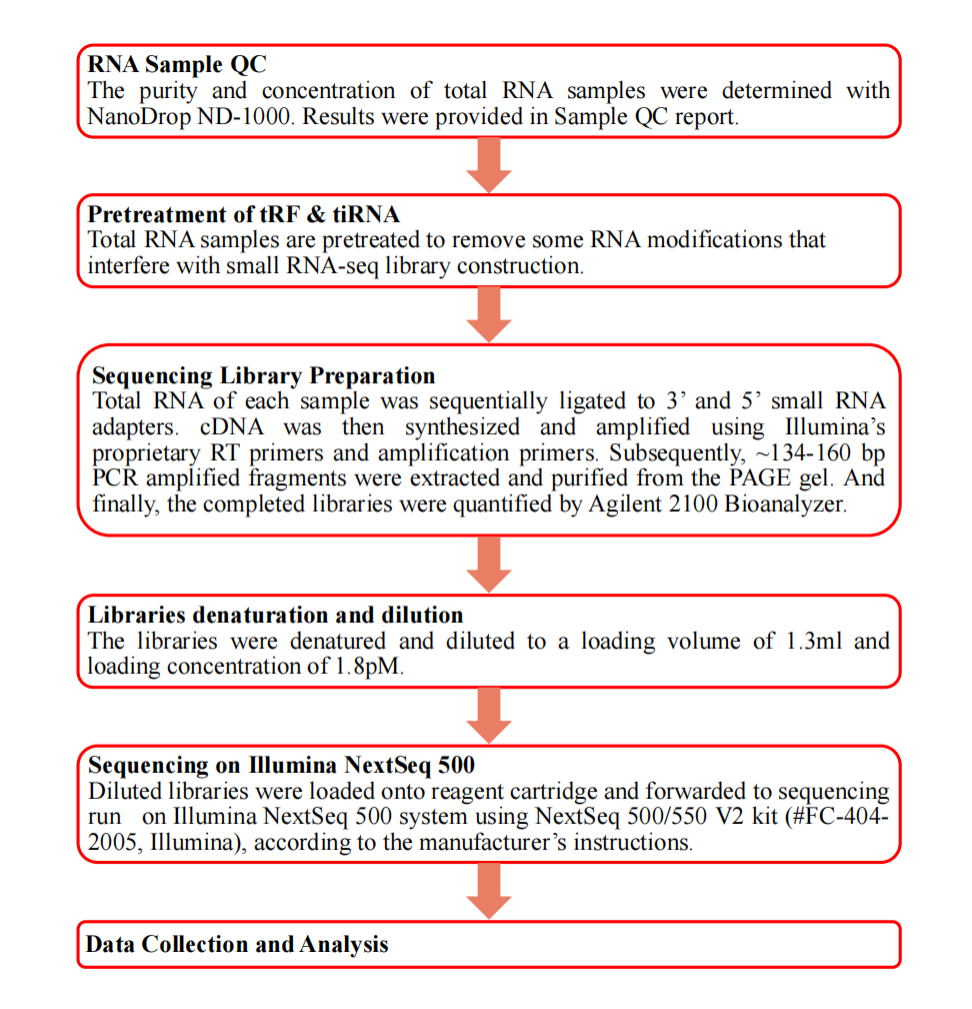

Supplement: Supplementary file 5 [file Image1.tif]

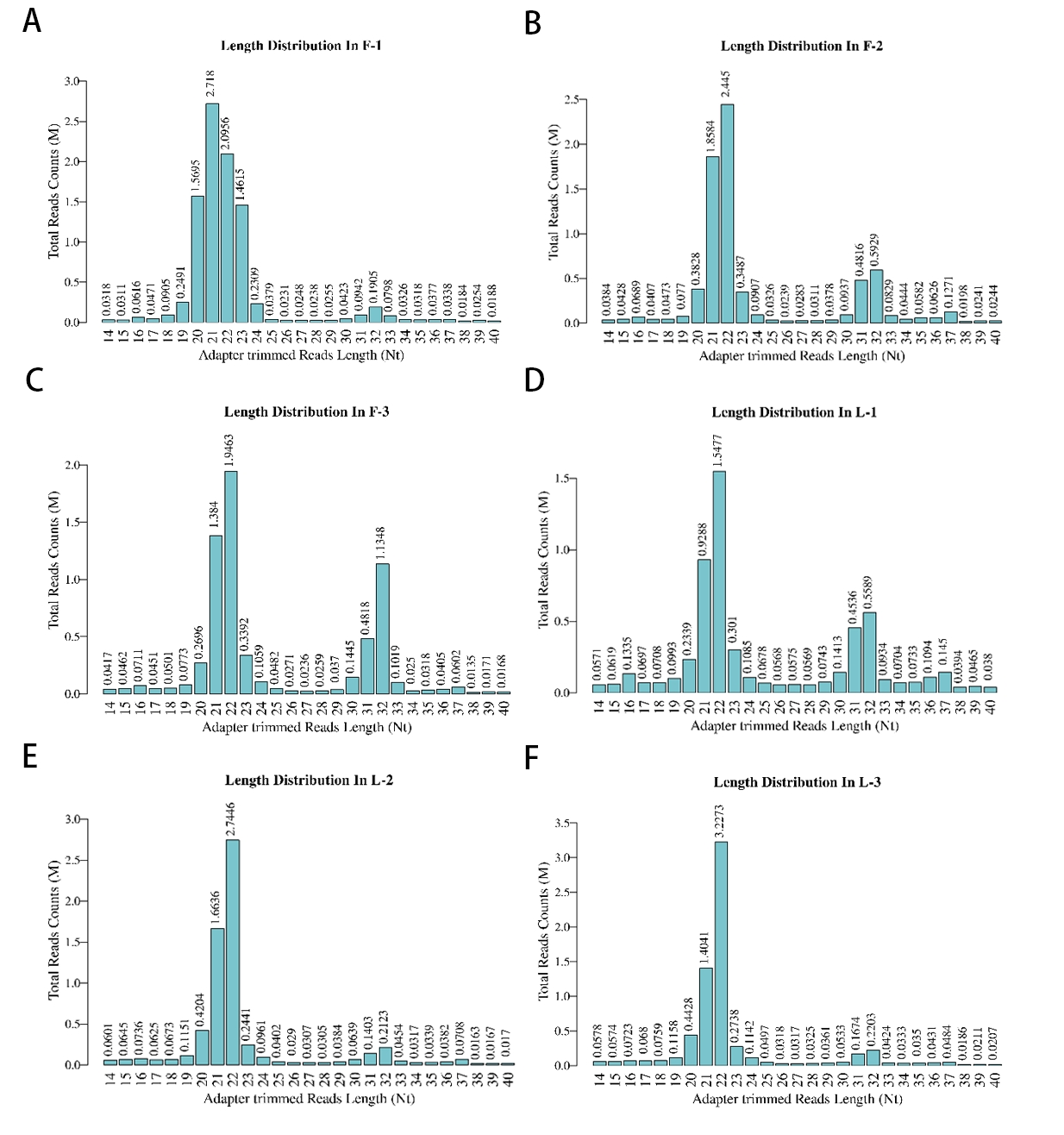

Supplement: Supplementary file 10 [file Image4.jpg]

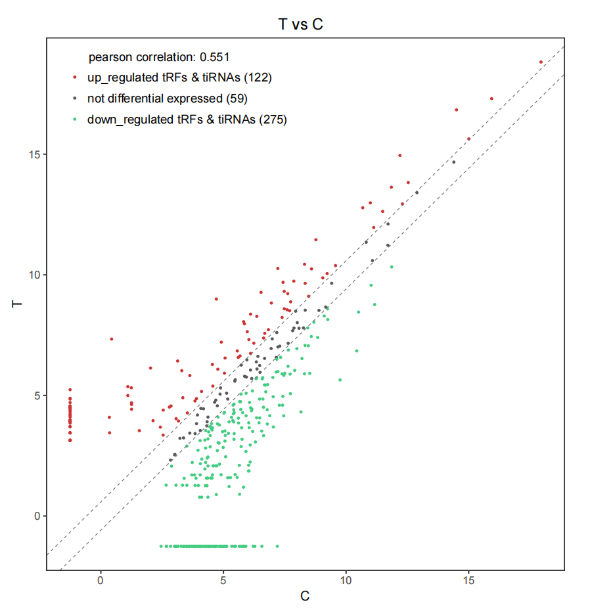

Supplement: Supplementary file 11 [file Image5.tif]
