# Supplementary material for: Expression profiles of exosomal tRNA-derived fragments and their biological functions in lipomas
Source: Front Cell Dev Biol. 2022 Aug 10;10:942133. doi: 10.3389/fcell.2022.942133 (PMC9399354; doi:10.3389/fcell.2022.942133)
Supplement: Supplementary file 3 [file Table1.docx]

| Sample | TotalRead | TotalBase | BaseQ30 | BaseQ30(%) |
| --- | --- | --- | --- | --- |
| F-1 | 10845250 | 542262500 | 482990697 | 89.07 |
| F-2 | 8715643 | 435782150 | 383110129 | 87.91 |
| F-3 | 7772132 | 388606600 | 347587102 | 89.44 |
| L-1 | 7609374 | 380468700 | 339785084 | 89.31 |
| L-2 | 7945238 | 397261900 | 353023322 | 88.86 |
| L-3 | 8405944 | 420297200 | 375934496 | 89.44 |
