# Supplementary material for: Expression profiles of exosomal tRNA-derived fragments and their biological functions in lipomas
Source: Front Cell Dev Biol. 2022 Aug 10;10:942133. doi: 10.3389/fcell.2022.942133 (PMC9399354; doi:10.3389/fcell.2022.942133)
Supplement: Supplementary file 6 [file Table2.docx]

| Sample | | Trimmed | | Mat-tRNA | | Mat-tRNA (%) | Pre-tRNA | Pre-tRNA(%) | miRNA | miRNA (%) | |
| --- | --- | --- | --- | --- | --- | --- | --- | --- | --- | --- | --- |
| F-1 | 9327115 | | 144004 | | 1.54 | | 7325 | 0.08 | 2643088 | 28.34 |  |
| F-2 | 7205833 | | 259238 | | 3.60 | | 10724 | 0.15 | 4551597 | 63.17 |  |
| F-3 | 6606113 | | 472313 | | 7.15 | | 7003 | 0.11 | 3436902 | 52.03 |  |
| L-1 | 5694950 | | 427255 | | 7.50 | | 10330 | 0.18 | 2557951 | 44.92 |  |
| L-2 | 6467291 | | 252976 | | 3.91 | | 8989 | 0.14 | 4647156 | 71.86 |  |
| L-3 | 6794894 | | 225204 | | 3.31 | | 8509 | 0.13 | 4818742 | 70.92 |  |
