# Supplementary material for: Expression profiles of exosomal tRNA-derived fragments and their biological functions in lipomas
Source: Front Cell Dev Biol. 2022 Aug 10;10:942133. doi: 10.3389/fcell.2022.942133 (PMC9399354; doi:10.3389/fcell.2022.942133)
Supplement: Supplementary file 7 [file Table3.docx]

| tRF_ID | | tRF_Seq | Type | Length |
| --- | --- | --- | --- | --- |
| tRF-58:76-Val-AAC-5 | | AAACCGGGCAGAAACACCA | tRF-3b | 19 |
| tRF-58: | 76-Val-CAC-2 | AAACCGGGCAGAAGCACCA | tRF-3b | 19 |
| tRF-58: | 76-Val-AAC- 1-M5 | AAACCGGGCGGAAACACCA | tRF-3b | 19 |
| tRF-52: | 71-chrM Pro-TGG | AAAGACTTTTTCTCTGACCA | tRF-3b | 20 |
| tRF-56: | 74-Pro-AGG- 1-M7- 18:C>A | AAATCCCGGACGAGCCCCA | tRF-3b | 19 |
