# Supplementary material for: Expression profiles of exosomal tRNA-derived fragments and their biological functions in lipomas
Source: Front Cell Dev Biol. 2022 Aug 10;10:942133. doi: 10.3389/fcell.2022.942133 (PMC9399354; doi:10.3389/fcell.2022.942133)
Supplement: Supplementary file 9 [file Table4.docx]

| tRF_ID | tRFdb_ID | p_value | F-1 | F-2 | F-3 | L-1 | L-2 | L-3 |
| --- | --- | --- | --- | --- | --- | --- | --- | --- |
| tiRNA-1:34-Val-CAC-1-M3 | - | 0.001814799 | 396.4871241 | 70.38027574 | 12.51783792 | 0 | 0 | 4.278752145 |
| tiRNA-1:33-Gly-CCC-2 | - | 0.001069768 | 1189.461372 | 200.0281521 | 87.62486543 | 43.42013552 | 38.17303838 | 4.278752145 |
| tiRNA-1:33-Gly-CCC-1 | - | 0.004234096 | 2022.084333 | 1048.295686 | 308.7733353 | 182.8216232 | 190.8651919 | 162.5925815 |
| tiRNA-1:33-Gly-GCC-2-M3 | - | 0.002702584 | 47076.23787 | 30563.5608 | 8355.656811 | 4789.926529 | 4256.29378 | 8429.141725 |
| tiRNA-1:34-Lys-CTT-1-M2 | - | 0.016771904 | 1328.231866 | 225.9577274 | 179.4223435 | 89.12554132 | 45.80764606 | 209.6588551 |
| tiRNA-1:33-Gly-GCC-1 | - | 0.006229339 | 4407.615196 | 2848.549055 | 529.9218052 | 365.6432465 | 381.7303838 | 924.2104633 |
| tRF-1:32-Gly-CCC-1-M4 | - | 0.008427378 | 145074.6387 | 123702.5952 | 40591.17576 | 18718.64895 | 24904.09024 | 45988.02805 |
| tiRNA-1:34-Lys-CTT-2 | - | 0.032586942 | 1400.921172 | 570.450656 | 271.2198216 | 70.843379 | 568.7782719 | 85.57504289 |
| tRF-+1:T20-Ser-TGA-1 | tRF-1001 | 0.03471933 | 2663.07185 | 770.4788081 | 406.8297324 | 235.3828399 | 393.1822953 | 607.5828046 |
| tiRNA-1:34-Glu-CTC-1-M2 | - | 0.023816891 | 14207.45528 | 3993.154592 | 1606.455866 | 3615.297599 | 194.6824958 | 1056.85178 |
| tRF-1:32-Glu-CTC-1-M2 | - | 0.030543875 | 5253.454394 | 12431.37923 | 2937.519298 | 2591.496509 | 1003.950909 | 3615.545562 |
| tRF-1:32-Gly-GCC-1 | - | 0.048152475 | 19308.92294 | 11283.06947 | 3782.473358 | 3535.313139 | 4752.543279 | 5622.280318 |
| tRF-57:76-Val-AAC-1-M5 | - | 0.001048198 | 0 | 0 | 0 | 36.56432465 | 343.5573454 | 243.8888722 |
| tRF-58:76-Val-AAC-1-M5 | - | 0.009330542 | 0 | 0 | 0 | 0 | 427.5380299 | 0 |
| tRF-1:28-Lys-TTT-3-M2 | - | 0.021012188 | 0 | 0 | 0 | 27.42324348 | 167.9613689 | 128.3625643 |
| tRF-59:77-Thr-AGT-2-M3 | - | 0.024916809 | 0 | 0 | 0 | 0 | 0 | 316.6276587 |
| tRF-1:30-Gln-CTG-1-M3 | - | 0.049444601 | 0 | 0 | 0 | 0 | 271.0285725 | 0 |
| tRF-57:75-Gln-CTG-1-M2 | - | 0.000415926 | 19.8243562 | 14.81690016 | 62.5891896 | 116.5487848 | 2049.892161 | 1583.138294 |
| tRF-1:31-iMet-CAT-1-M2 | - | 0.006418994 | 0 | 40.74647543 | 6.25891896 | 185.1068935 | 797.8165022 | 175.4288379 |
| tRF-1:15-Val-AAC-1-M9 | - | 0.000951114 | 85.90554355 | 66.6760507 | 104.315316 | 265.0913537 | 2637.756952 | 3020.799014 |
| tRF-1:16-Tyr-GTA-4-M4 | - | 0.039506198 | 19.8243562 | 29.63380031 | 0 | 0 | 572.5955757 | 209.6588551 |
| tRF-1:29-Glu-TTC-1 | - | 0.038545743 | 13.21623747 | 22.22535023 | 79.27964015 | 553.0354103 | 725.2877293 | 0 |
| tRF-59:75-Gln-CTG-1-M5 | tRF-3004a | 0.014930535 | 436.1358365 | 451.9154548 | 212.8032446 | 658.1578436 | 3924.188346 | 5083.157548 |
| tRF-1:15-Thr-CGT-5 | - | 0.040802619 | 337.0140555 | 348.1971537 | 187.7675688 | 1014.660009 | 2706.468421 | 2058.079782 |
